# Inverted Organic Solar Cells with Low-Temperature Al-Doped-ZnO Electron Transport Layer Processed from Aqueous Solution

**DOI:** 10.3390/polym10020127

**Published:** 2018-01-28

**Authors:** Qianni Zhang, Ruizhi Peng, Chunfu Zhang, Dazheng Chen, Zhenhua Lin, Jingjing Chang, Jincheng Zhang, Yue Hao

**Affiliations:** 1 Wide Bandgap Semiconductor Technology Disciplines State Key Laboratory, School of Microelectronics, Xidian University, Xi’an 710071, China; qiannizhang93@outlook.com (Q.Z.); 18257560789@163.com (R.P.); zhlin@xidian.edu.cn (Z.L.); jjingchang@xidian.edu.cn (J.C.); jchzhang@xidian.edu.cn (J.Z.); yhao@xidian.edu.cn (Y.H.); 2 Shaanxi Joint Key Laboratory of Graphene, Xidian University, Xi’an 710071, China

**Keywords:** organic solar cell, inverted structure, electron transport layer, Al-doped-ZnO

## Abstract

The aqueous-based Zn-ammine complex solutions represent one of the most promising routes to obtain the ZnO electron transport layer (ETL) at a low temperature in inverted organic solar cells (OSCs). However, to dope the ZnO film processed from the Zn-ammine complex solutions is difficult since the introduction of metal ions into the Zn-ammine complex is a nontrivial process as ammonium hydroxide tends to precipitate metal salts due to acid-base neutralization reactions. In this paper, we investigate the inverted OSCs with Al-doped-ZnO ETL made by immersion of metallic Al into the Zn-ammine precursor solution. The effects of ZnO layer with different immersion time of Al on film properties and solar cell performance have been studied. The results show that, with the Al-doped-ZnO ETL, an improvement of the device performance could be obtained compared with the device with the un-doped ZnO ETL. The improved device performance is attributed to the enhancement of charge carrier mobility leading to a decreased charge carrier recombination and improved charge collection efficiency. The fabricated thin film transistors with the same ZnO or AZO films confirm the improved electrical characteristics of the Al doped ZnO film.

## 1. Introduction

Organic solar cells (OSCs) based on composites of conjugated polymers (electron donor) and fullerene derivatives (electron acceptor) have attracted more and more attention due to their potential of low cost, light-weight physical features and commercial large area production [1,2,3,4,5,6,7]. With persistent efforts, the power conversion efficiency (PCE) of OSCs has been greatly improved to be above 10% in the past decade [2,8,9,10,11,12,13,14,15,16,17,18,19]. OSCs are mainly fabricated with two structures: the conventional structure and the inverted structure. In a typical conventional structure, the indium tin oxide (ITO) and the low-work-function metal (e.g., Al, Ca) are usually used as the transparent anode and opaque cathode, and an active layer (donor–accepter phase-separated blend) is sandwiched between the anode and cathode. However, the long-term stability is a problem because of the degradation of a low-work-function cathode by oxygen and water vapor and the corrosion of ITO by acidic and hygroscopic poly(3,4-ethylenedioxithiophene):poly(styrene sulfonate) (PEDOT:PSS), which is used as the hole transport layer [20,21,22,23]. To solve this problem, inverted OSCs have been developed with an improvement in stability and the capability for the roll-to-roll fabrication process. Compared with the conventional structure, inverted OSCs utilize ITO as the cathode and air-stable high-work-function metals like Ag as the anode [24,25,26,27].

In inverted OSCs, an n-type mental oxide as the electron transport layer (ETL) is introduced between the ITO cathode and the active layer instead of PEDOT:PSS, which improves the device stability. N-type metal oxides such as aluminum oxide (Al_2_O_3_), titanium oxide (TiO_x_), and zinc oxide (ZnO) have been extensively investigated [28,29,30,31,32,33]. In particular, ZnO has been viewed as a promising candidate as a result of several advantages such as low-cost, high transparency in visible region and stable oxidation. To obtain a high performance inverted OSC, a high conductivity ETL is required. However, the intrinsic conductivity of the ZnO film processed at a low temperature is still low. Therefore, developing a high performance ZnO thin film is a key issue. As reported previously [34,35,36,37,38], the doped ZnO film is found with better properties such as higher charge carrier mobility. In solar cells, the LiF doped and Li doped ZnO have been reported, and both of them were shown to enhance the charge collection efficiency and reduce the charge carrier recombination, resulting in a higher photovoltaic performance [34,35]. In the meantime, the aluminum doped and other metals’ (e.g., Ba, Mg and Sr) doped ZnO films have been introduced in organic light-emitting diodes, leading to higher charge carrier mobility [36,37,38]. 

The aqueous-based Zn-ammine complex solutions represent one of the most promising routes to obtain the ZnO film at a low temperature due to the extremely low decomposition temperature [39,40,41,42]. This makes it possible to deposition the ZnO thin-film onto flexible substrates [43,44]. However, very different from the sol-gel method processed ZnO (where to dope the ZnO is relatively easy), to dope the ZnO film processed from the Zn-ammine complex solutions is difficult since the introduction of metal ions into the Zn-ammine complex is a nontrivial process as ammonium hydroxide tends to precipitate metal salts due to acid–base neutralization reaction. Recently, it has been shown that the immersion of metallic Al into the Zn-ammine precursor solution for different amounts of time is an easy and efficient way to dope the ZnO film, which has been used in thin film transistors (TFTs) [45]. However, the Al doped ZnO (AZO) processed by this method has never been used in inverted OSCs.

In this paper, we investigate the inverted OSCs based on the poly(3-hexylthiophene): phenyl-C61-butyric acid methyl ester (P3HT:PC_61_BM) blend system and the poly({4,8-bis[(2-ethylhexyl)oxy]benzo[1,2-b:4,5-b′]dithiophene-2,6-diyl}{3-fluoro-2-[(2-ethylhexy)carbonyl]thieno[3,4-b]thiophenediyl}):[6,6]-phenyl-C71-butyric acid methyl ester (PTB-7:PC_71_BM) blend system with the immersion of metallic Al into the Zn-ammine precursor solution processed AZO as the ETL. The effects of ZnO layer with different immersion times of Al on film properties and solar cell device performance have been studied. The results show that, when Al has an appropriate immersion time in the ZnO solution, an improvement of the device performance could be obtained compared with the device with the un-doped ZnO ETL because of the enhancement of charge carrier mobility leading to a decreased charge carrier recombination and improved charge collection efficiency. We also fabricate and investigate TFTs with the ZnO film and the results confirm the improved electrical characteristics of the Al doped ZnO film.

## 2. Materials and Methods

### 2.1. Materials

All the materials, zinc oxide (99.9%, Sigma-Aldrich, Saint Louis, MI, USA), ammonia solution (≥28%, NH_3_ in H_2_O, Aladdin, Hamden, CT, USA), poly(3-hexylthiophene) (P3HT, BASF), phenyl-C_61_-butyric acid methyl ester (PC_61_BM, 98%, Nano-C, Westwood, MA, USA), poly({4,8-bis[(2-ethylhexyl)oxy]benzo[1,2-b:4,5-b′]dithiophene-2,6-diyl}{3-fluoro-2-[(2-ethylhexy)carbonyl]thieno[3,4-b]thiophenediyl}) (PTB-7, 1-material), [6,6]-phenyl-C71-butyric acid methyl ester (PC_71_BM, 99%, Nano-C), 1,8-diiodooctane (DIO, 98%, Sigma-Aldrich), 1,2-dichlorobenzene (99%, Sigma-Aldrich), chlorobenzene (99.8%, Sigma-Aldrich), molybdenum oxide (MoO_3_, 99.98%, Sigma-Aldrich) are used as received without further purification.

For the low-temperature aqueous solution processing, ZnO solution was obtained by ZnO powder directly dissolved in ammonia solution (8 mg/mL). For Al-doped-ZnO (AZO), Al was added into well prepared ZnO solution with different immersion times. 

### 2.2. Film Formation and Inverted Solar Cells Fabrication

Inverted OSCs were fabricated on pre-patterned ITO glass substrates (around 2 × 2.5 cm^2^ in size, 10 Ω per square, surface roughness of 2–3 nm). The patterned ITO glass substrates were sequentially cleaned with detergent, de-ionized water, acetone, alcohol and isopropyl alcohol in an ultrasonic bath at 50 °C for 20 min, respectively. Then, the cleaned ITO substrates were dried with nitrogen and treated in a UV ozone oven for 15 min. After that, on the top of the ITO substrates, the ZnO solution was spin-coated at 3000 rpm for 45 s, and thermally annealed in the baking oven at 150 °C for 30 min. Subsequently, the substrates were transferred into a nitrogen-filled glovebox. The active layer solution used P3HT and PC_61_BM blend with a weight ratio of 1:1 in 1,2-dichlorobenzene(1,2-DCB) (20 mg/mL). The blend solution was spin-coated on the ZnO layer at 800 rpm for 120 s, and the active layer was annealed at 150 °C for 15 min. Finally, the devices were finished by thermal evaporation of 8 nm MoO_3_ layer and 80 nm Ag electrode. The device area is 7 mm^2^. 

For OSCs based on the PTB-7:PC_71_BM blend system, the same device fabrication procedures were used except the spin coating of the PTB-7 and PC_71_BM blend (weight ratio of 2:3 in chlorobenzene with 3 vol % DIO) at 1000 rpm for 60 s, and the following dried in the shade at least for 5 h.

The TFTs were fabricated with the same ZnO solution used in solar cells. Before the film fabrication, the cleaned Si/SiO_2_ (100 nm) wafer should be treated with O_2_ plasma for 10 min to remove the surface residues and facilitate the thin film formation. The ZnO solution was spin-coated on the wafer at 3000 rpm for 30 s, annealed at 300 °C for 5 min, and then spin-coated a second time with the same condition. Finally, a 100 nm Al layer was deposited on the top of ZnO to form the source and drain contacts.

### 2.3. Device Characterization

The photovoltaic performances of OSCs were measured by using a Keithley 2400 source meter (Tektronix, Inc., OR, USA)under a simulated AM 1.5G sunlight from XES-70S1 solar simulator (XES-301, SEN-EI Electric. Co. Ltd, Osaka, Japan) with an intensity of 100 mW/cm^2^. The system was calibrated against a National Renewable Energy Laboratory (NREL) certified silicon reference solar cell. Incident photo-to-electron conversion efficiency (IPCE) was measured under short-circuit conditions by a solar cell quantum efficiencies system (SCS10-X150, Zolix instrument. Co. Ltd., Beijing, China) with a monochromatic light from an arc lamp. 

The surface morphologies of the ZnO films and the active layers deposited on different ZnO films were characterized by an atomic force microscopy (AFM, Bruker Dimension Icon, Bruker, Karlsruhe, Germany). The UV-visible absorption spectra were recorded with an UV-visible spectrophotometer (Perkin-Elmer Lambda 950, Waltham, MA, USA). All of the above measurements were performed under ambient atmosphere at room temperature without encapsulation.

## 3. Results and Discussion

**Figure 1:**
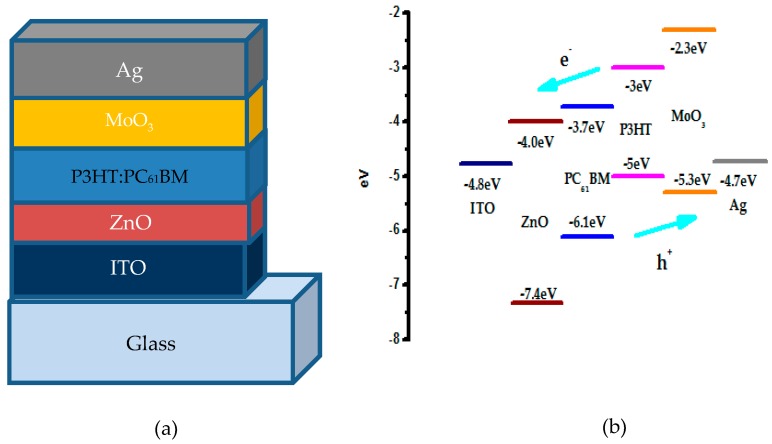
(**a**) Schematic poly(3-hexylthiophene): phenyl-C61-butyric acid methyl ester (P3HT:PC_61_BM) device structure used in this paper. The thickness of each layer is not in scale with the real thickness for clarity; (**b**) Schematic illustration of the energy levels of the component materials of the studied devices.

The schematic device structure and the energy level diagram of the component materials of the inverted P3HT:PC_61_BM OSCs are shown in Figure 1a,b. In this paper, we use the inverted device structure of ITO/ZnO(AZO)/active layer/MoO_3_/Ag. From Figure 1b, the conduction band minimum of ZnO is approximately 4.0 eV, which is closed to the lowest unoccupied molecular orbital (LUMO) of PC_61_BM, leading to a facilitated electron transport to ITO cathode, since the valance band maximum of ZnO is anticipated at 7.4 eV, which will effectively block the hole from the highest occupied molecular orbital (HOMO) of P3HT. 

**Figure 2:**
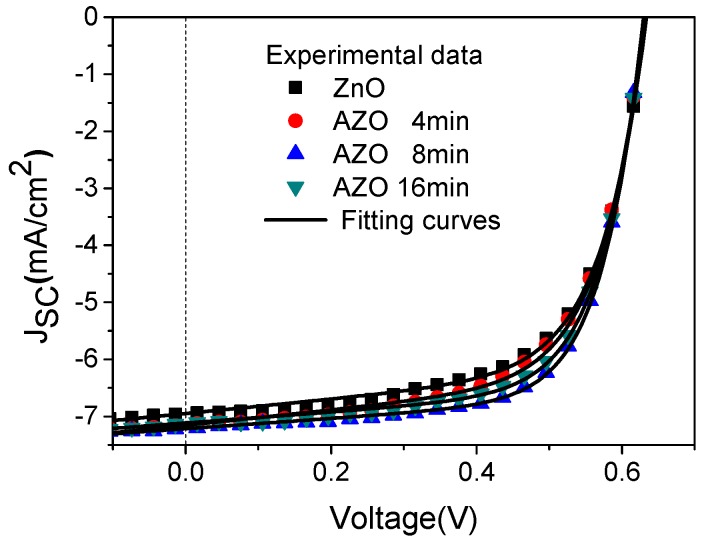
J-V characteristics of the P3HT:PC_61_BM devices introducing the ZnO film without and with the different immersion times of Al doping.

**Figure 3:**
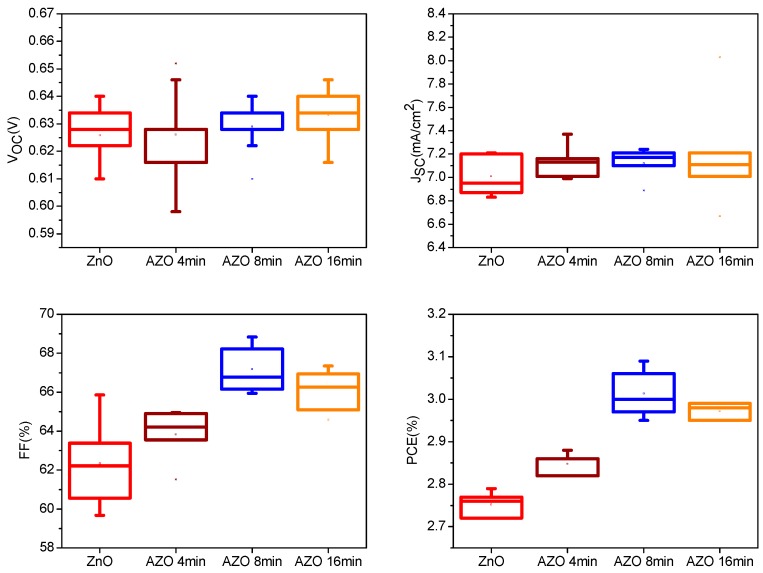
The statistical results of the photovoltaic parameters of the inverted organic soalr cells (OSCs) incorporating the ZnO layer without and with the different Al immersion time. Every statistical result is derived from more than 20 devices.

Figure 2 illustrates the current density vs. voltage (J-V) characteristics of the inverted OSCs introducing the ZnO buffer layer un-doped and doped with different amounts of Al by controlling different immersion times. The parameters of OSCs are extracted according to the Shockley equation:

(1) $$J=J_{0}(\exp(\frac{q(V-R_{s}J)}{nK_{B}T})-1)+\frac{V-R_{s}J}{R_{sh}}-J_{p},$$

where *J*_0_ is the saturation current, *J_p_* the photocurrent, *R_s_* the series resistance, *R_sh_* the shunt resistance, *n* the ideality factor, *q* the electron charge, *k_B_* the Boltzmann constant, and *T* the temperature. By using Equation (1) with our proposed explicit analytic expression method [46], the experimental data were extracted and these parameters could rebuild the I-V curves of the OSCs as shown in Figure 2, which confirmed the validity of the extracted parameters. The photovoltaic performance parameters of the best devices are summarized in Table 1. The device based on the pure ZnO buffer layer shows a short-circuit current density (J_SC_) of 6.95 mA/cm^2^ and a fill factor (FF) of 63.38%. With Al immersion time form 4 min to 16 min, both of them have an obvious increase. The increased FF may be due to the reduced charge recombination and increased shunt resistance (*R_sh_*). In particular, when the Al immersion time is 8 min, the device shows the optimized performance with J_SC_ of 7.21 mA/cm^2^ and FF of 68.21%. Therefore, PCE of the device increases from 2.79% to 3.09%. However, when the immersion time is increased to 16 min, there shows a slight decrease in J_SC_, FF and PCE. Since the J_SC_ value is related to the properties of the ETL. It is inferred that when the Al immersion time is short, the doped Al could improve the charge carrier mobility and thus enhance the device performance. However, when the Al immersion time is long, the excess Al will become the scattering center, which will partly decrease the charge carrier mobility. This will be confirmed by the following charge carrier mobility measurement. Figure 3 shows the statistical results of the dependence of open circuit voltage (V_OC_), FF, J_SC_ and PCE on the immersion time of Al in the ZnO solution. From the statistical results, it could be seen that the device with the Al immersion time at 8 min shows the best performance, which confirms the validity of the above discussion. From Figure 3, it is found that V_OC_ slightly increases after doping Al into the ZnO solution from 4 min to 16 min. It corresponds to the previous report that V_OC_ of the device increases with the increase of Al doping amount due to the Fermi energy shift of AZO film [47]. Meanwhile, V_OC_ is related to the photon energy loss. The smaller photon energy loss occurred in the device may be caused by the reduced interface related recombination or energy loss, which will result in a higher V_OC_. 

**Figure 4:**
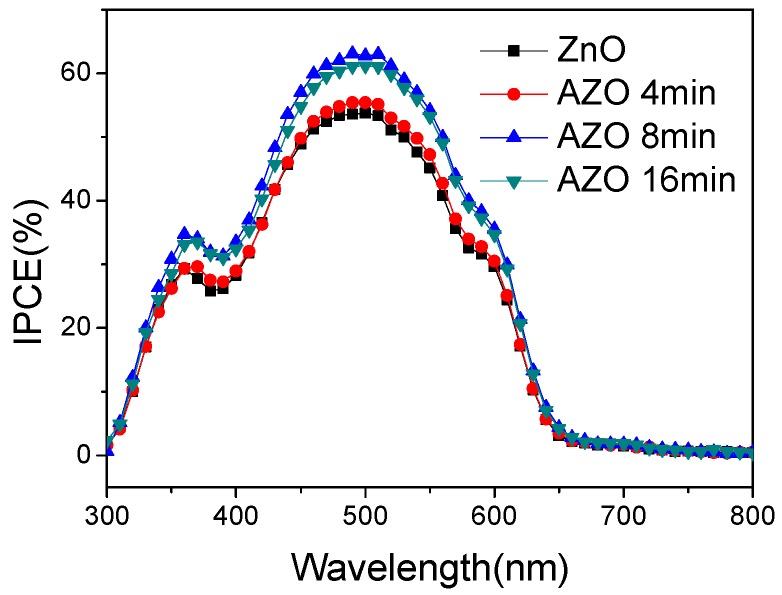
Incident photon-to-current conversion efficiency (IPCE) spectra of inverted P3HT:PC_61_BM solar cells.

Incident photon-to-current conversion efficiency (IPCE) spectra of the ZnO (AZO)/P3HT:PC_61_BM devices are presented in Figure 4. Compared with the maximum IPCE 53.7% of the device with the pure ZnO ETL, the devices with the 8 min and 16 min AZO buffer layers have the maximum IPCE of 61.18% and 63.07% at the wavelength around 500 nm, respectively. The integrated IPCE value of device with 8 min AZO thin film is 7.42 mA/cm^2^, which is close to the measured J_SC_. The IPCE measurement results also confirm that the device with the 8 min AZO buffer layer has the best performance.

**Figure 5:**
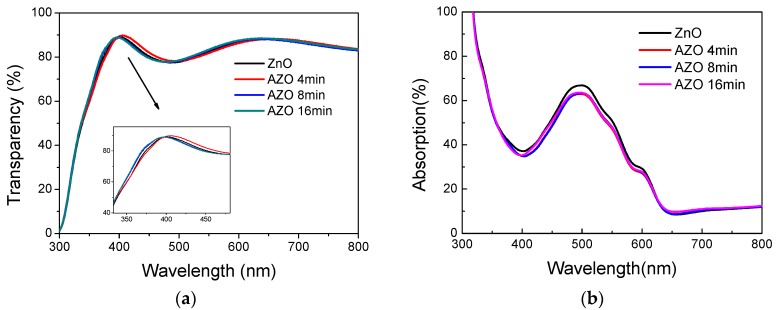
(**a**) The transmittance spectra of the ZnO the electron transport layer (ETL); (**b**) The absorption spectra of the active layer (P3HT:PC_61_BM) with difference Al immersion times.

The transmittance spectra of the ZnO ETL and the absorption spectra of the active layer (P3HT:PC_61_BM) with difference Al immersion times are shown in Figure 5a,b. As can be seen, all of the ZnO/ITO films have similar good transmittance in the visible wavelength range (from 300 nm to 800 nm). It indicates that doping Al into the ZnO solution has a minimal effect on the transmittance of the ZnO thin film. As shown in Figure 5b, the absorption of the active layer with the un-doped ZnO layer is a bit higher at the wavelength from 400 nm to 600 nm. This shows that the introduction of Al in the ETL could slightly reduce the absorption ability of the active layer. Although the mechanism behind this is still not known, it indicates that the improvement of the device performance with the AZO ETL is due to the improved electrical properties of the AZO layer instead of the improved light absorption.

**Figure 6:**
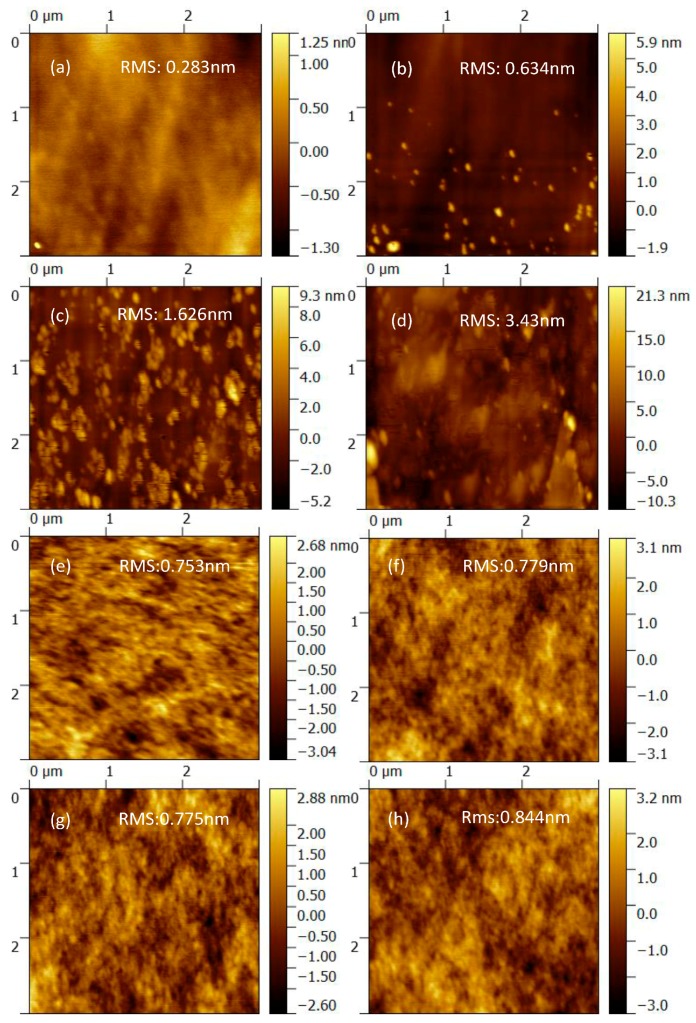
(**a**–**d**) Surface morphologies of ZnO thin film; (**e**–**h**) Surface morphologies of ZnO(AZO)/P3HT:PC_61_BM, without or with Al immersion time of 4 min, 8 min and 16 min, respectively.

In order to further investigate the ZnO/AZO films, the surface morphologies of them and P3HT:PC_61_BM active layers were measured by the atomic force microscopy (AFM). The results are illustrated in Figure 6. For the increase of the immersion time of Al in ZnO solution, the topography images (Figure 6a–d) changes significantly. The surface of the AZO film is rougher than the pure ZnO film and the root-mean-square (rms) values of the surface roughness increases from 0.283 nm to 3.430 nm for the Al immersion time from 0 min to 16 min. On one side, the rougher ETL increases the contact area with active layer, from which the electron collection may benefit and lead to an increased J_SC_. On the other side, if the roughness is too large, when voltage on the device is added, it is easier to cause the device breakdown. Combing the measured device performance in Figure 2 and Figure 3, the immersion time of 8 min is optimal. Compared with obvious changes on the surface of the ZnO film, the surface morphologies of the P3HT:PC_61_BM active layer with the ZnO or AZO ETL almost remain unchanged. The result indicates that the surface morphology of the ZnO buffer layer almost has a minimal effect on the topography of active layer. It is inferred that other factors such as the electrical properties could affect the device performance. 

**Figure 7:**
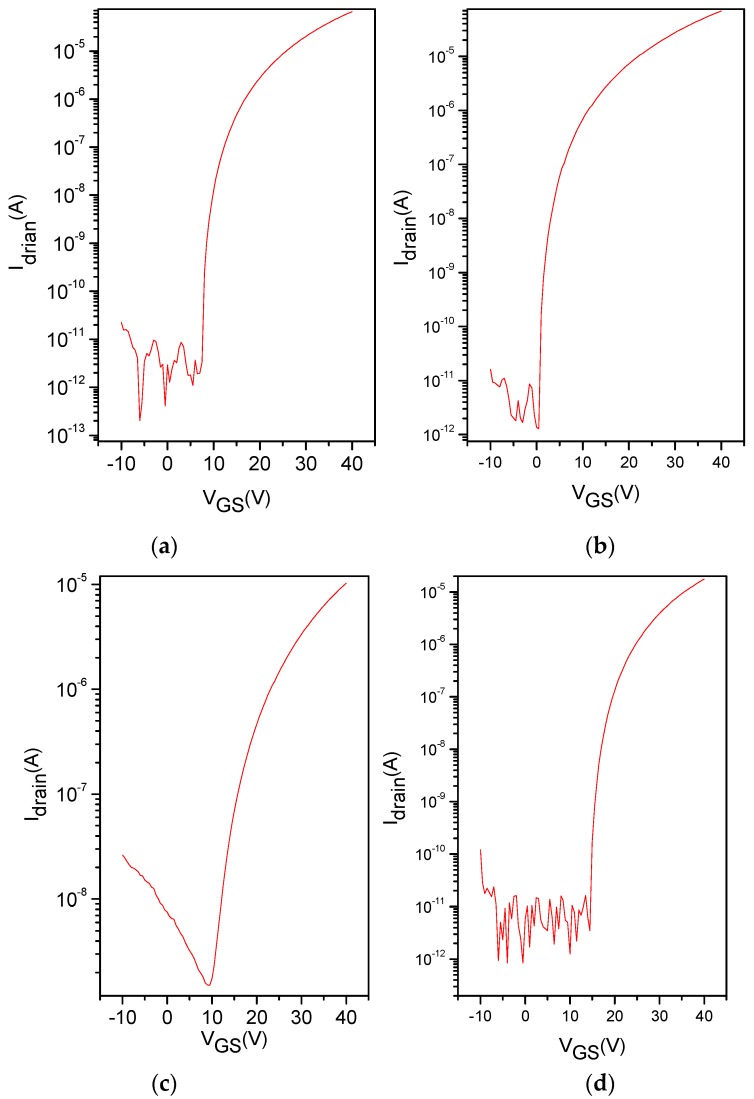
Transfer characteristics of Al doped ZnO thin film transistors (TFTs) with different reaction times (0 min (**a**), 4 min (**b**), 8 min (**c**) and 16 min (**d**)).

To evaluate the electrical properties of the ZnO/AZO films, the transistors are fabricated. The transfer characteristics for the ZnO TFTs with different Al immersion times are shown in Figure 7. The results show that TFTs fabricated with the un-doped ZnO film exhibit an electron mobility of 0.18 cm^2^ V^−1^ s^−1^, and with the increase of the Al immersion time, the electron mobility increases to 0.95 cm^2^ V^−1^ s^−1^ (8 min). As shown in Figure 7, the drain current increases by doping Al into ZnO. However, the current begins to decrease when the immersion time is further increased to 16 min. This is consistent with the performance change of OSCs. The results above indicate that an appropriate amount of Al doped into ZnO solution can improve the properties of the film, resulting in better performance in both TFTs and inverted OSCs.

**Figure 8:**
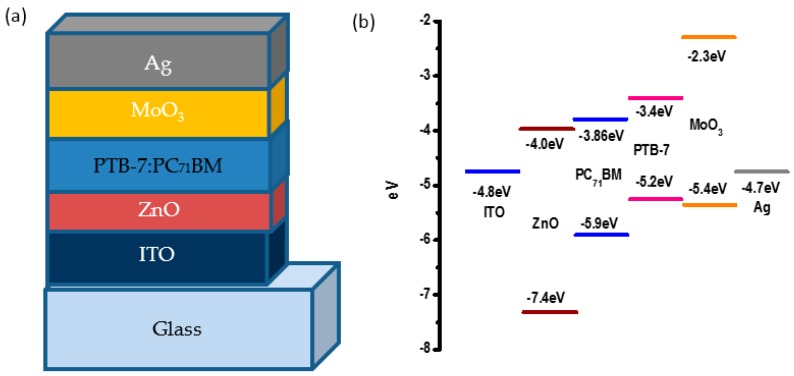
(**a**) Schematic device structure with PTB-7:PC_71_BM. The thickness of each layer is not on scale with the real thickness for clarity; (**b**) Schematic illustration of the energy levels of the component materials of PTB-7:PC_71_BM devices.

To further confirm the validity of using Al doped ZnO as ETL to improve the device performance, the PTB-7:PC_71_BM OSCs were also fabricated. Figure 8 shows the corresponding schematic device structure and the energy level diagram of the component materials. The structure is similar to the P3HT:PC_61_BM OSCs except for the active layer. 

**Figure 9:**
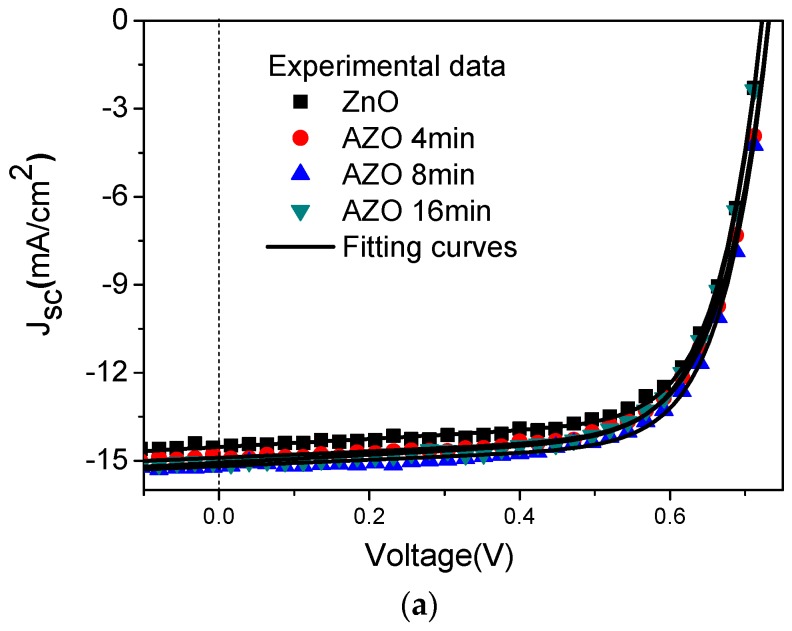

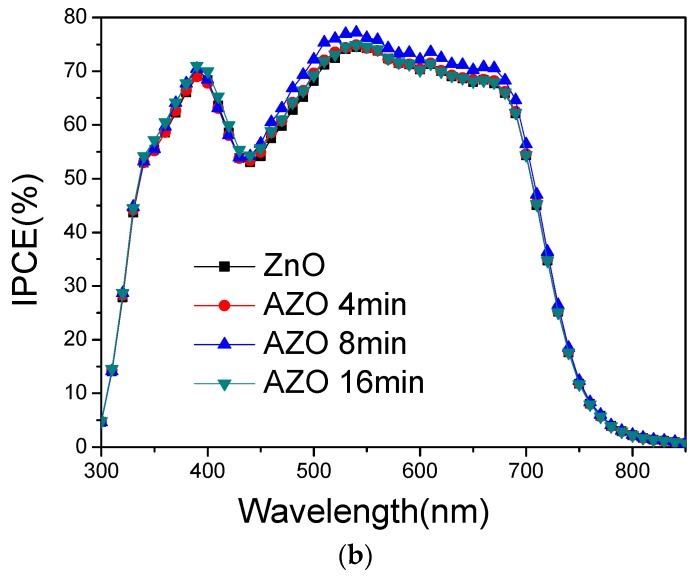
(**a**) J-V characteristics of the PTB-7:PC_71_BM devices introducing the ZnO film without and with the different immersion times of Al doping; (**b**) IPCE spectra of the corresponding PTB-7:PC_71_BM solar cells.

**Figure 10:**
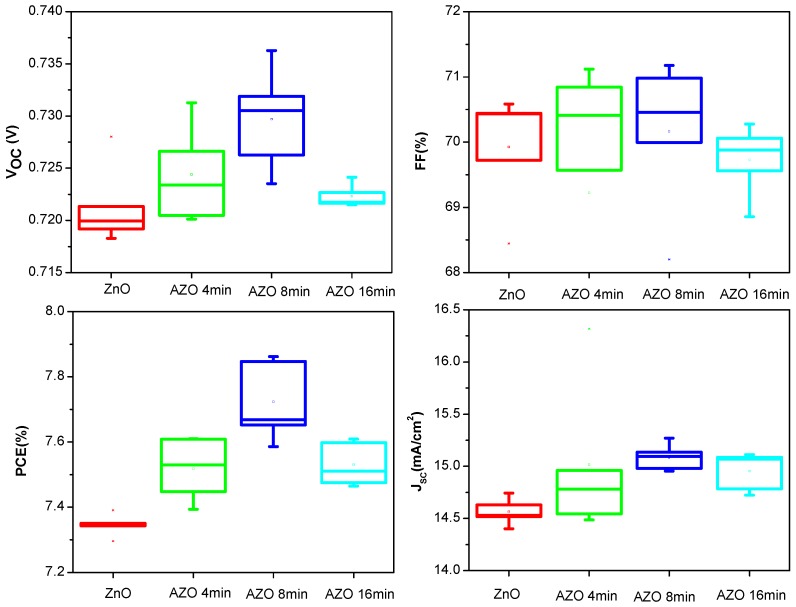
The statistical results of the photovoltaic parameters of the inverted PTB-7:PC_71_BM OSCs incorporating the ZnO layer without and with the different Al immersion times. Every statistical result is derived from more than 20 devices.

The photovoltaic performance parameters of the best PTB-7:PC_71_BM devices are summarized in Table 2, and the J-V curves are shown in Figure 9. From the parameters, it is shown that the PCE of the device with AZO as ETL is higher than that with ZnO (7.39%), especially the AZO device with immersion time of Al at 8 min (7.86%). Furthermore, in the PTB-7:PC_71_BM system, using proper AZO can also improve the V_OC_ and FF. The IPCE measurement results in Figure 9b also show that the device with the Al immersion time of 8 min achieves the highest value, which corresponds to the best device performance. The statistical results of the photovoltaic parameters in Figure 10 further confirmed the validity of above discussion. 

## 4. Conclusions

In summary, Al-doped-ZnO (AZO) thin film as the ETL in inverted OSCs has been fabricated via spin coating aqueous solution, which is a low temperature processing. Using AZO (immersion time 8 min) as the buffer layer in P3HT:PC_61_BM system, PCE increases to 3.09%, compared with the control device with a pure ZnO device (PCE 2.79%) under the same conditions. Furthermore, in a PTB-7:PC_71_BM system, PCE with AZO (immersion time 8 min) can be improved to be 7.86% (pure ZnO, PCE 7.39%). Our result suggests that AZO with this simple doping method can improve the performance of inverted OSCs.

## Figures and Tables

**Table 1:** Photovoltaic parameters of best inverted structure P3HT:PC_61_BM solar cells with ZnO or Al-doped-ZnO (AZO) as the electron transport layer ETL.

Device	V_OC_ (V)	J_SC_ (mA/cm^2^)	FF (%)	PCE (%)
ZnO	0.634	6.95	63.38	2.79
AZO 4 min	0.628	7.13	64.21	2.88
AZO 8 min	0.628	7.21	68.21	3.09
AZO 16 min	0.628	7.11	66.94	2.99

**Table 2:** Photovoltaic parameters of best inverted structure PTB-7:PC_71_BM solar cells with ZnO or AZO as the ETL.

Device	V_OC_ (V)	J_SC_ (mA/cm^2^)	FF (%)	PCE (%)
ZnO	0.721	14.52	70.58	7.39
AZO 4 min	0.731	14.96	69.57	7.61
AZO 8 min	0.732	15.13	70.98	7.86
AZO 16 min	0.723	15.06	69.88	7.60
